# Antimicrobial activity screening of rhizosphere soil bacteria from tomato and genome-based analysis of their antimicrobial biosynthetic potential

**DOI:** 10.1186/s12864-020-07346-8

**Published:** 2021-01-07

**Authors:** Lu Zhou, Chunxu Song, Zhibo Li, Oscar P. Kuipers

**Affiliations:** 1 Department of Molecular Genetics, University of Groningen, Groningen, The Netherlands; 2 National Academy of Agriculture Green Development, Key Laboratory of Plant-Soil Interactions, Ministry of Education, College of Resources and Environmental Sciences, China Agricultural University, Beijing, 100193 China

**Keywords:** *Bacillus*, Plant growth promoting Rhizobacteria (PGPR), Genome mining, Tomato, Rhizosphere, Biosynthetic gene clusters, Non-ribosomal synthesized peptides, Polyketides, Bacteriocins

## Abstract

**Background:**

Tomato plant growth is frequently hampered by a high susceptibility to pests and diseases. Traditional chemical control causes a serious impact on both the environment and human health. Therefore, seeking environment-friendly and cost-effective green methods in agricultural production becomes crucial nowadays. Plant Growth Promoting Rhizobacteria (PGPR) can promote plant growth through biological activity. Their use is considered to be a promising sustainable approach for crop growth. Moreover, a vast number of biosynthetic gene clusters (BGCs) for secondary metabolite production are being revealed in PGPR, which helps to find potential anti-microbial activities for tomato disease control.

**Results:**

We isolated 181 *Bacillus*-like strains from healthy tomato, rhizosphere soil, and tomato tissues. In vitro antagonistic assays revealed that 34 *Bacillus* strains have antimicrobial activity against *Erwinia carotovora*, *Pseudomonas syringae; Rhizoctonia solani*; *Botrytis cinerea*; *Verticillium dahliae* and *Phytophthora infestans*. The genomes of 10 *Bacillus* and *Paenibacillus* strains with good antagonistic activity were sequenced. Via genome mining approaches, we identified 120 BGCs encoding NRPs, PKs-NRPs, PKs, terpenes and bacteriocins, including known compounds such as fengycin, surfactin, bacillibactin, subtilin, etc. In addition, several novel BGCs were identified. We discovered that the NRPs and PKs-NRPs BGCs in *Bacillus* species are encoding highly conserved known compounds as well as various novel variants.

**Conclusions:**

This study highlights the great number of varieties of BGCs in *Bacillus* strains. These findings pave the road for future usage of *Bacillus* strains as biocontrol agents for tomato disease control and are a resource arsenal for novel antimicrobial discovery.

**Supplementary Information:**

The online version contains supplementary material available at 10.1186/s12864-020-07346-8.

## Background

Tomato (*Solanum lycopersicum*) is the second most important vegetable crop worldwide after potato, based on the sizes of their growth areas [1]. However, tomato crops face serious threats of disease, partially due to the use of cultivars susceptible to diseases that are causing substantial production losses [2]. The overuse of chemical pesticides has contaminated soils and has caused harmful effects on human beings [3]. Accordingly, putting biocontrol agents isolated from nature into the soil is environmentally friendly and useful for tomato crop disease control. One way to improve plant growth is by using plant growth-promoting rhizobacteria (PGPR), since PGPR have the ability to colonize the roots and express their plant growth promotion activities in the rhizosphere [4].

The rhizosphere, a narrow zone of soil that surrounds and is influenced by plant roots, gives home to an overwhelming variety of organisms, in particular microorganisms such as bacteria, fungi, oomycetes, archaea, protozoa and algae [5, 6]. This complex microbial community has profound effects on plant growth since it facilitates nutrient absorption and provides health protection to plants [7]. Among all the microorganisms, PGPR has been largely described for their biocontrol capabilities. They can promote plant growth either indirectly by suppression of diseases with secreted antimicrobials or directly by the improvement of physiological metabolic processes such as N_2_ fixation, phosphate solubilization and IAA production [8].

Among PGPR, the group of Gram-positive *Bacillus* strains has been studied less intensively, compared to widely used Gram-negative bacteria, like *Pseudomonas* strains [9]. One of the most efficient Gram-positive bacteria that promote plant growth belongs to the genus *Bacillus*. *Bacillus subtilis* is used in agriculture to protect plants from several plant pathogens since it can either indirectly protect plants by inducing systemic resistance (ISR) against a broad range of pathogens or directly excrete antimicrobials [10–13]. Besides, *Bacillus* species can produce hard, resistant endospores to allow them to resist adverse environmental conditions and permit easy formulation and storage of the commercial products [14].

The *Bacillus* species offer a plethora of antagonistic compounds displaying a broad range of biological functions, which have good potential to be used as biocontrol agents for tomato disease control [15]. All the bioactive secondary metabolites are encoded by biosynthetic gene clusters (BGCs). Based on their products, BGCs are classified as ribosomally synthesized peptides (linear bacteriocin BGCs and ribosomally-produced an posttranslationally modified peptides [RiPPs]), non-ribosomally synthesized peptides synthetases (NRPSs) BGCs and polyketide synthases (PKSs) BGCs [16]. Here, we set out to find novel BGCs in *Bacillus* strains which encode potentially active compounds to inhibit plant-pathogens. Based on genome mining, 10 selected (out of 351) promising *Bacillus* strains newly isolated from rhizosphere soils of healthy tomato plants and tissues were characterized with respect to anti-pathogen activities. Subsequently, several novel BGCs were discovered, which have potential functions in tomato pathogens antagonism.

## Results

### Isolation of Bacteria and in vitro antagonistic assay

**Fig. 1:**
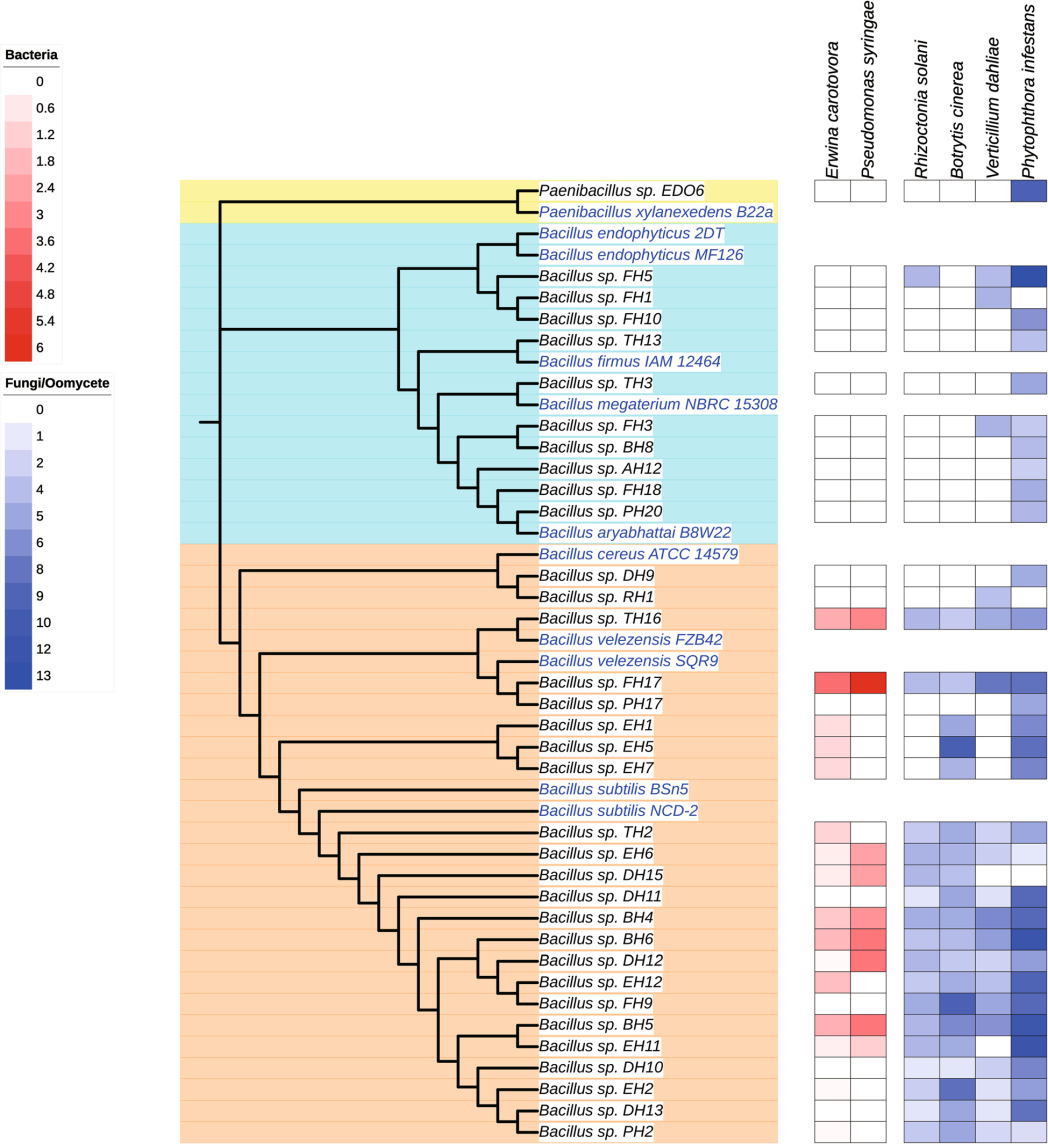
Neighbor-joining tree based on 16S rRNA genes of 34 isolated strains showing antagonistic activity against tomato plant bacterial, fungal and oomycetal pathogens. Red represents the inhibition halo size of bacterial pathogens and blue indicates the inhibition halo size of fungal and oomycetal pathogens. The red and blue scale bar represent the radius of inhibition halo observed (mm)

**Fig. 2:**
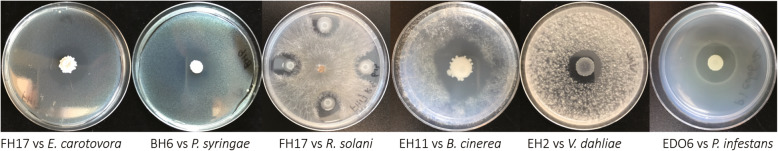
Pictures of antagonistic assay for each kind of pathogens in plates

A total of 181 *Bacillus*-like strains were isolated from healthy tomato rhizosphere soil and tomato plant tissue collected in either the Netherlands or Spain. Among them, 28 endophytic strains were isolated from healthy tomato plant tissues collected in Spain. 74 and 79 rhizosphere bacteria strains were isolated from tomato plants collected in Spain and the Netherlands, respectively. In order to identify potential PGPR strains, all the *Bacillus*-like strains were preliminarily screened by in vitro antagonistic activity against six major tomato plant pathogens, i.e. *Erwinia carotovora* [17], *Pseudomonas syringae* [18], *Rhizoctonia solani* [3], *Botrytis cinerea* [19], *Verticillium dahliae* [20]*,* and *Phytophthora infestans* [21]. The results revealed that 34 *Bacillus*-like strains could inhibit different bacterial, fungal and oomycetal plant pathogens growth on plates (Figs. 1 and 2).

We found that 34 *Bacillus*-like strains were distributed between three large major clusters of the neighbor-joining tree based on 16S rRNA genes. In the first cluster, strain EDO6 was clustered within the genus of *Paenibacillus*, which was related to type strain *Paenibacillus xylanexedens* B22a, with the percent identity value above 99.6% on 16S rRNA gene sequence. Ten isolated strains were grouped in the second cluster and were closely related to the type strains *Bacillus endophyticus* 2DT and MF126, *Bacillus firmus* IAM 12464, *Bacillus megaterium* NBRC 15308, or *Bacillus aryabhattai* B8W22. The third cluster consisted of 23 isolated strains. They were tightly releated to reference strains *Bacillus cereus* ATCC 14579, *Bacillus velezensis* FZB42 or SQR9, *Bacillus subtilis* BSn5 and NCD-2.

Of all strains, seven strains (TH16, FH17, EH6, DH15, BH4, BH5 and BH6) showed inhibition on all pathogens. Among them, four strains (TH16, FH17, BH5 and BH6), showing the biggest inhibition halo on all pathogens, were selected to sequence their genomes for further research. In addition, three strains (EH11, EDO6 and FH5), showing the highest inhibition activity (inhibition halo size > 9.75 mm against *P. infestans*), were also selected for genome sequencing. Besides, two strains (EH2 and EH5), showing the highest inhibition activity (inhibition halo size > 8.5 mm) against *B. cinerea*, were selected for genome sequencing as well. Strain DH12 was also selected for genome sequencing, because of the large inhibition halo size (> 3.38 mm) measured on the plates against *Pseudomonas syringae*. In summary, a total of 10 strains (BH5, BH6, DH12, EH2, EH5, EH11, FH5, FH17, TH16 and EDO6) were genome sequenced for further research.

### Genome sequencing and phylogenetic analysis

**Fig. 3:**
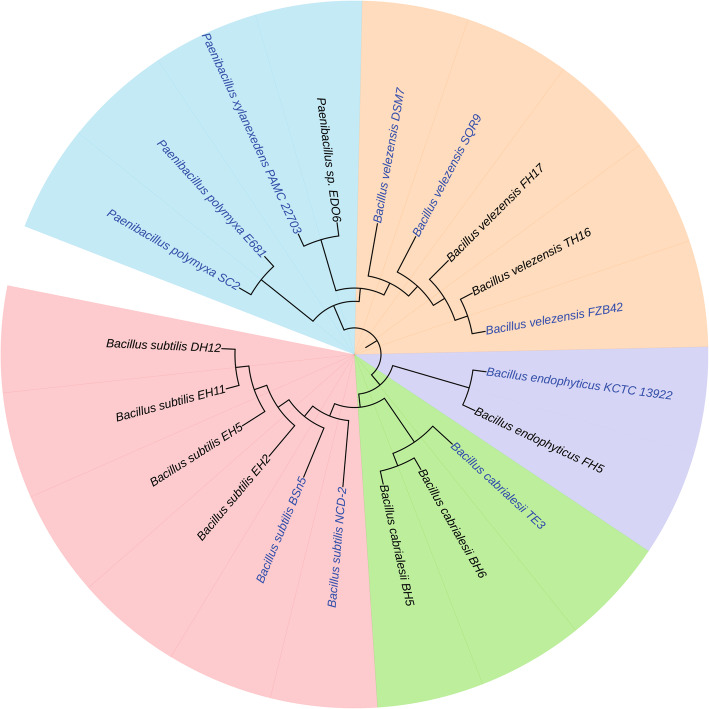
Phylogenetic position of 10 isolated *Bacillus* and *Paenibacillus* strains with high significant antagonistic activity against tomato pathogens. A maximum likelihood (ML) tree was constructed based whole genome sequences analysis using Gegenee

The genomes of 10 isolated strains were sequenced, assembled and annotated as described in a previous study [22]. Based on whole genome phylogenetic analyses, the 10 *Bacillus* strains were clustered into five clades as presented in Fig. 3. All of them were tightly clustered together with reported PGPR strains from the *Bacillus* class, such as *B. subtilis* Bsn5, *B. velezensis* FZB42 and *P. polymyxa* E681. This suggests that they probably can promote plant growth as well, which needs to be further investigated. Moreover, to classify strains at the species level, Average Nucleotide Identity (ANI) and digital DNA-DNA Hybridization (dDDH) values were determined [23] (Additional file 1: Table S1). Strains DH12, EH2, EH5, and EH11 were exhibiting ≥ 98.21% ANI and ≥ 86.70% dDDH compared with reference genome *B. subtilis* Bsn5, therefore they were identified as *B. subtilis* species. Strains FH17 and TH16 were identified as *B. velezensis, based on* ≥ 98.14% ANI and ≥ 85.30% dDDH compared with reference genome of *B. velezensis* FZB42. Strains BH5 and BH6 were classified into *B. cabrialesii* species because of exhibiting 96.60% ANI and 73.40% dDDH compared with the reference genome of *B. cabrialesii* TE3*.* Strain FH5 was identified as *B. endophyticus* based on 96.35% ANI and 73.40% dDDH compared with reference genome *B. endophyticus* KCTC 13922. Strain EDO6 could not be classificated at the species level due to the low ANI and dDDH values (93.88% ANI and 57.60% dDDH), even compared with the closest species *P. xylanexedens* PAMC 22703, so we will name it *Paenibacillus* sp. EDO6.

### Biosynthesis gene cluster (BGC) mining

A total of 120 BGCs were found, averaging 12 clusters per genome. All the BGCs were designated as those encoding NRPSs, PKSs, terpenes, hybrid NRPS/PKSs, bacteriocins, RiPPs and others (Table 1 and Additional file 1: Table S2). The BGCs encoding surfactin [24], fengycin [24], bacillibactin [25], subtilosin A [26], bacillaene [27], macrolactin [28], difficidin [29], and subtilin [30] were discovered in the genomes. Besides, some BGCs encoding unknown compounds, were also identified (Table 1). Most of the unknown BGCs (76.47%) are PKSs BGCs, which cannot be assigned to any known compounds. 73.07% bacteriocins BGCs encodes potential novel peptides. 27.78 and 27.27% of NRPSs and Hybrids BGCs are still unknown. These findings provide a great opportunity of new bioactive compounds discovery.

**Table 1:** Distribution of BGC totals in 10 isolated strains (A) and percentages of BGCs encoded unknown compounds identified from genome sequence (B)

**A.**								
**Strains**		**Predicted BGCs**	**NRPS**	**PKS**	**Hybrid NRPS/PKS**	**Terpene**	**Bacteriocin**	**Other**
*Bacillus cabrialesii* BH5		12	4	1	1	2	3	1
*Bacillus cabrialesii* BH6		12	4	1	1	2	3	1
*Bacillus subtilis* DH12		12	4	1	1	2	3	1
*Bacillus subtilis* EH2		10	3	1	1	2	2	1
*Bacillus subtilis* EH5		11	3	1	1	2	3	1
*Bacillus subtilis* EH11		12	4	1	1	2	3	1
*Bacillus endophyticus* FH5		10	2	1	1	2	3	1
*Bacillus velezensis* FH17		15	5	4	1	2	1	2
*Bacillus velezensis* TH16		12	4	4	1	1	1	1
*Paenibacillus* sp. EDO6		14	3	2	2	1	4	1
**B**								
**BGC Types**	**Total BGCs**	**% Unknown**	**Known compounds**					
NRPSs	36	27.78	surfactin (8 BGCs), fengycin (8 BGCs), bacillibactin (10 BGCs)					
PKSs	17	76.47	macrolactin (2 BGCs), difficidin (2 BGCs)					
Hybrids	11	27.27	bacillaene (8 BGCs)					
Bacteriocin	26	73.07	subtilin (2 BGCs), subtilosin A (6 BGCs)					

### Novel NRPs and PKs BGCs identified from the 10 strains

**Fig. 4:**
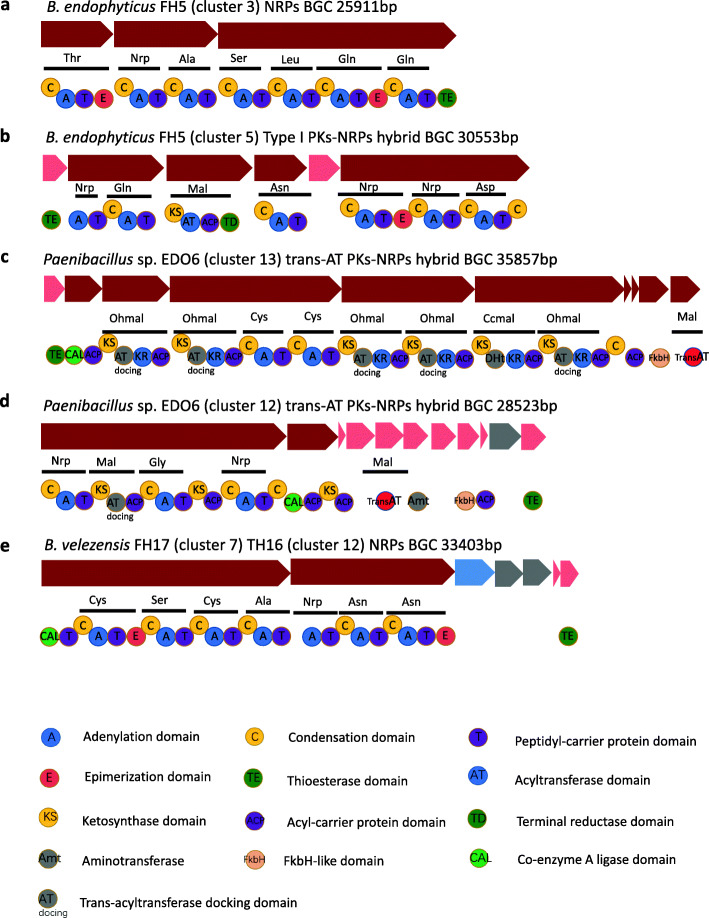
Novel Biosynthetic Gene Clusters (BGCs) identified from the isolated *Bacillus* and *Paenibacillus* strains. **a** an NRPs BGC discovered in *B. endophyticus* FH5. **b** a Type I PKs-NRPs hybrid BGC found in *B. endophyticus* FH5. **c**, **d** two trans-AT PKs-NRPs hybrid BGCs harboered by *Paenibacillus sp.* EDO6. **e** an NRPs BGC found in both *B. velezensis* FH17 and TH16

The majority of BGCs could be assigned to known compounds, whereas 5 clusters represented probably novel NRPs and PKs-NRPs hybrid BGCs for which no or low similarity BGCs could be identified in the MIBiG [31] database (Fig. 4).

Two novel gene clusters were identified from *B. endophyticus* FH5. One NRPs (Fig. 4a) BGC consists of three genes and has a total size of 25 kb. Three genes are encoding 24 domains, which includes 7 condensation (C) domains, 7 adenylation (A) domian, 7 thiolation (T) domain, 2 epimerization (E) domain and 1 thioesterase (TE) domain. All the domains are essential components in this BGC and catalyze primary formation of a lipopeptide product. This BGC is showing no similarity to any known BGCs reported. The other one (Fig. 4b) is a Type I PKs-NRPs hybrid BGC with a size of approximately 30 kb. The PKs module consists of a ketosynthase (KS) domain, a acyltransferase (AT) domain, an acyl carrier protein (ACP) domain and a terminal reductase (TD) domain. It likely incorporates the polyketide moiety of malonyl-CoA, while the NRPs modules incorporate six amino acid residues. Based on antiSMASH analysis, only 28% genes show similarity to the known paenilamicin BGC. Paenilamicin [32], synthesized by *pam* BGC from *Paenibacillus larvae* DSM25430, has antibacterial and antifungal activity. The *pam* cluster consists of five NRPs genes, two Type I PKs genes, and two Type I PKs-NRPs hybrid genes, and has a size of ∼60 kb. In contrast, the Type I PKs-NRPs hybrid BGC identified in *B. endophyticus* FH5 consists of only three NRPS genes and one Type I PKS gene. All of them differ from the *pam* cluster of *Paenibacillus larvae* DSM25430.

In the genome of *Paenibacillus* sp. EDO6, two novel trans-AT PKs-NRPs hybrid gene clusters (cluster 13 and cluster 12) were discovered, which have the sizes of almost 35 kb and 28 kb, respectively (Fig. 4c and d). The order and domain of the genes of both hybrid clusters differ from each other. Specifically, Cluster 13 has an additional dehydratase domain variant (DHt) playing an important role during polyketide biosynthesis through the dehydration of the nascent polyketide intermediate to provide olefins [33], which cannot be found in cluster 12. In addition to the differences observed at the domain level of core biosynthetic genes, regulator and transporter genes are also different. Moreover, only 33 and 21% of the genes of cluster 13 and cluster 12 exhibit similarity to known pellasoren and xenocoumacin BGCs respectively. Pellasoren [34] was isolated from myxobacterium, which has shown to possess potential anti-cancer activity. The known pellasoren BGC, is a Type I PKs-NRPs hybrid cluster identified from *Sorangium cellulosum* So ce38 and consists of six genes of Type I PKs and one single gene of NRPs as compared to the trans-AT PKs-NRPs hybrid gene (cluster 13) of *Paenibacillus* sp. EDO6, which in turn consists of four trans-AT PKs genes and one trans-AT PKs-NRPs hybrid gene. Xenocoumacin [35] is the main anti-bacterial and anti-fungal compound produced by *Xenorhabdus nematophila*. The known xenocoumacin BGC, also being a Type I PKs-NRPs hybrid cluster, which was identified from *Xenorhabdus nematophila* ATCC 19061, consists of four genes of Type I PKs and two genes of NRPs whereas cluster 12 from *Paenibacillus* sp. EDO6 consists of one single trans-AT domain gene, one gene of trans-AT PKs and one gene of trans-AT PKs-NRPs hybrid.

One novel NRPs BGC was discovered both in *B. velezensis* FH17 and TH16 (Fig. 4e). This BGC contains seven genes with a size of approximately 33 kb. Whereas seven modules are only encoded by two core biosynthetic genes, seven amino acids are incorporated into the final product. This BGC shows no similarity to any known clusters. Furthermore, a single heterocyclization (Cy) domain in the first module is found.

### Novel Ribosomally synthesized and post-translationally modified peptides (RiPPs) identified in the 10 strains

**Fig. 5:**
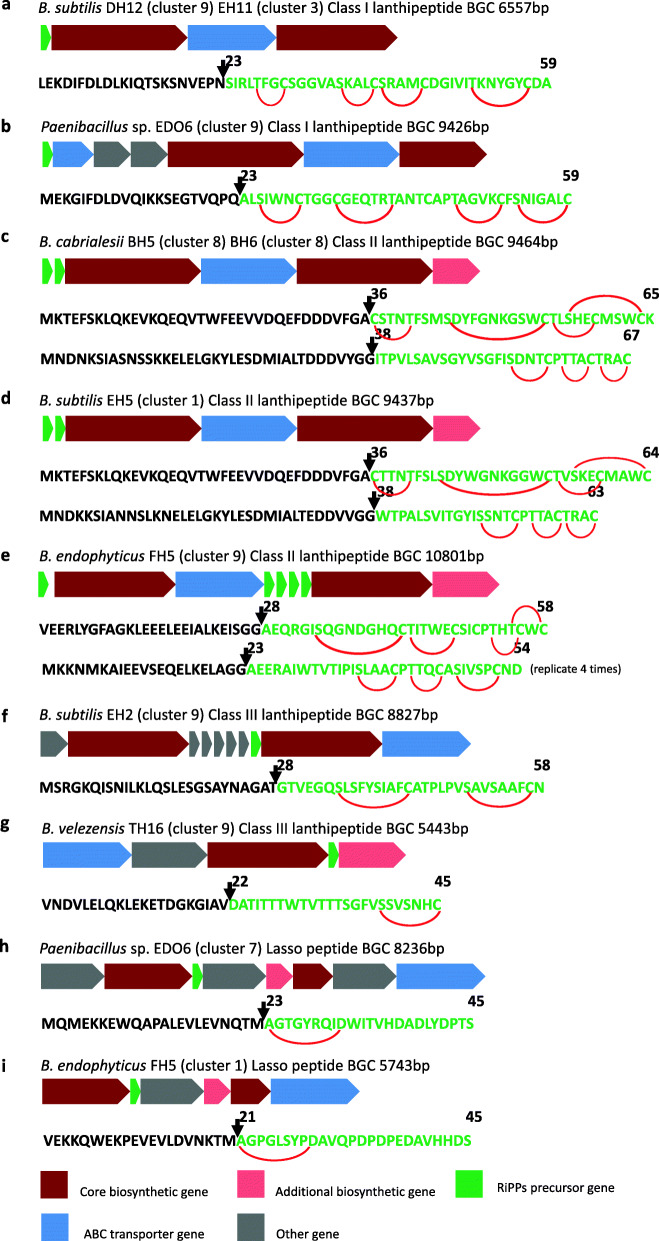
Novel bacteriocin Biosynthetic Gene Clusters (BGCs) identified from the isolated *Bacillus* and *Paenibacillus* strains. **a**, **b** two different class I lanthipeptide BGCs discovered in *B. subtilis* DH12 and *Paenibacilus* sp. EDO6 respectively. **c** class II lanthipeptide BGC found in both *B. cabrialesii* BH5 and BH6. **d**, **e** two different class II lanthipeptide BGCs discovered in *B. subtilis* EH5 and *B. endophyticus* FH5 respectively. **f**, **g** two different class III lanthipeptide BGCs found in *B.subtilis* EH2, *B. velezensis* TH16 respectively. **h** two different lasso peptide BGCs discovered in *Paenibacillus* sp. EDO6 and *B. endophyticus* FH5 respectively

A total of nine novel bacteriocin BGCs were identified from the 10 strains (Fig. 5). All of them are belong to RiPPs (less than 10 kDa). These peptides are ribosomally synthesized, and undergo posttranslational modifications (PTMs), resulting in different structures and properties, mainly showing anti-bacterial activity against closely related producer strains [36].

Two novel gene clusters were identified as class I lanthipeptide BGCs. One lanthipeptide BGC was identified from both *B. subtilis* DH12 and EH11 with a size of ∼6 kb (Fig. 5a). This BGC consists of four genes. The precursor peptide contains 59 amino acids, which shows no similarity to any known bacteriocins. Another one lanthipeptide BGC (Fig. 5b) was identified from *Paenibacillus* sp. EDO6 with a size of ∼9 kb. This BGC contains seven genes. The precursor peptide encoded by the core biosynthetic gene contains 59 amino acids, which also shows no similarity to any known bacteriocins.

Three novel BGCs were identified as class II lanthipeptide BGCs. All of them belong to two-component lanthipeptides consisting of two peptides. The individual peptides of two-component lanthipeptides only have little or no antimicrobial activity, but the two peptides act in synergy to exhibit significantly higher activity in equimolar concentrations [37]. Both *B. cabrialesii* BH5 and BH6 harbor the same two-component lanthipeptide BGC (Fig. 5c). It consists of six genes with a size of ∼9 kb. This BGC has 70% of genes showing similarity to staphylococcin C55 α/β BGC [38]. The presursors of two core biosynthetic genes (α and β) of this BGC identified contain 65 and 67 amino acids respectively. The C terminus (from C36 to K65) of the α precursor is belonging to the plantaricin C family of lantibiotics with a identity of 83.33% to the known peptide staphylococcin C55 α. Whereas the C terminus (from I38 to C67) of the β precursor shows 62.07% identity to lacticin 3147 A2 [39]. The second novel class II lanthipeptide BGC was discovered from *B. subtilis* EH5 (Fig. 5d). This BGC has six genes with a length of ∼9 kb. The presursors of two core peptide genes (α and β) contain 65 and 67 amino acids respectively. It is also showing 70% gene sequence similarity to staphylococcin C55 α/β BGC. The C terminus (from C36 to C64) of the α precursor has a similarity of 79.31% to the known peptide staphylococcin C55 α and the C terminus (from W38 to C63) of the β precursor is showing 72% identity to lacticin 3147 A2. The third BGC was identified from *B. endophyticus* FH5 (Fig. 5e). It is comprised of nine genes with a size of ∼10 kb. Its precursors of two peptides (α and β) contain 58 and 54 amino acids respectively. There is no similarity found to any known BGCs. The C terminal region (from A28 to C58) of the α precursor has a similarity of 53.33% to the known peptide plantaricin W α [40] and the C terminus (from A23 to D54) of the β precursor is showing 56.25% identity to haloduracin β [41]. Furthermore, the precursor β in this potential novel BGC found in *B. endophyticus* FH5 has four replicates, indicating potential high amount production of β peptide.

Two novel gene clusters were identified as class III lanthipeptide BGCs. This Class contains RiPPs that are modified by the mutifunctional enzymes LanKC. LanKC firstly phosphorylates the Ser/Thr residuses in the substrate peptide and then similarly catalytizes modification of the substrate to form the final product, as the class II lanthipeptide LanM enzyme [42]. The one identified from *B. subtilis* EH2 contains ten genes with a size of ∼8 kb (Fig. 5f). No similarity was found to any known BGCs. The full precursor contains 58 amino acids. The predicted cleaveage site by antiSMASH is between T27 and G28. The C terminus (from G28 to N58) of the precursor has no identity to any known RiPPs. The other class III lanthipeptide BGC is harbored by *B. velezensis* TH16 (Fig. 5g). This one contains five genes with a length of ∼5 kb. The core biosynthetic gene encodes a 45-amino acid precursor peptide. 35% genes of this BGC show similarity to locillomycin [43], which is a cyclic lipopeptide (NRPs) discovered from *B. subtilis* 916. The predicted cleaveage site is between V21 and D22 by antiSMASH and the C terminus (from D22 to C45) of the precursor has no identity to any known RiPPs.

Two novel lasso peptide BGCs were identified from the genomes of *Paenibacillus* sp. EDO6 and *B. endophyticus* FH5. The one from *Paenibacillus* sp. EDO6 contains eight genes with a size of ∼8 kb (Fig. 5h). It shows that gene sequences are 60% similar to that of the paeninodin BGC [44]. The precursor peptide contains 45 amino acids. The predicted cleaveage site is between M22and A23. The core peptide (from A23 to S45) shows 33.3% identity to the paeninodin [44] from *P. dendritiformis* C454. Another novel lasso peptide BGC was mined from *B. endophyticus* FH5 (Fig. 5i). This BGC comprised of six genes. It is showing 80% genes similarity to paeninodin. Its precursor peptide contains 45 amino acids. The cleaveage site is between M20 and A21. The core peptide (from A21 to S45) has 76% identity to the paeninodin.

### Large-scale genome-based analysis of the bioactive potential of *Bacillus*

Lipopetides produced by the *Bacillus* genus are involved in the biocontrol mechanisms of plant pathogens [45]. To gain a general overview of BGCs distributed in the genomes of *Bacillus* genus, the diversity of BGCs in the genomes of *Bacillus* isolated was investigated. A total of 9459 BGCs were predicted and identified, which included NRPs (2377 BGCs), RiPPs (1564 BGCs), Type I PKs (517 BGCs), PKs-NRPs hybrids (309 BGCs), PKs (including Trans AT-PKs and Type III PKs) (1369 BGCs), Terpene (970 BGCs), Saccharide (62 BGCs) and Others (2291 BGCs).

**Fig. 6:**
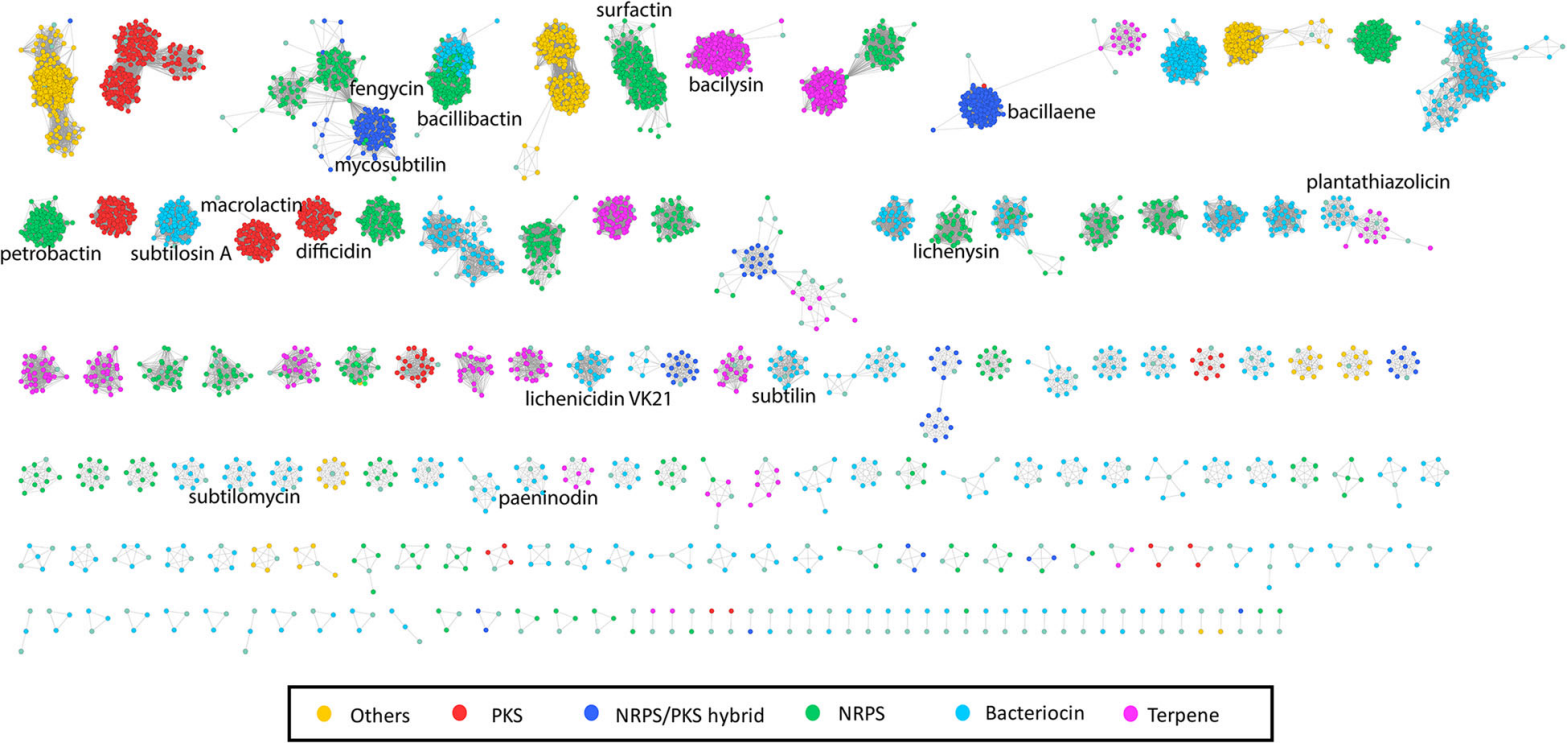
The predicted Biosynthetic Gene Clusters (BGCs) Similarity network of 555 *Bacillus* strains genomes showing their diversity, distribution. The lines between the nodes represent genes shared between BGCs

The similarity network of predicted BGCs revealed that a large number of BGCs are present in *Bacillus* strains, and are distributed throughout different kinds of secondary metabolites (Fig. 6). Based on our investigation, some of the NRPs BGCs were conserved among the BGCs identified in the *Bacillus* species. 259 out of 2377 (10.85%) NRPs BGCs were encoding surfactin, 330 (13.88%) BGCs were encoding bacillibactin, 110 (4.63%) NRPs BGCs were encoding fengycin, 158 (6.65%) NRPs BGCs were encoding petrobactin [46]. And 38 (1.60%) NRPs BGCs were encoding lichenysin [47]. Thus, a total of ∼38% of the NRPs BGCs are correlated to already reported compounds. Additionally, most of PKs-NRPs hybrid BGCs (67.64%) were ecoding bacillaene. Unlike the well-described NRPs and PKs-NRPs hybrid BGCs, the PKs BGCs were mostly attributed to unknown products with the exception of macrolactin [48] and difficidin [29]. Notably, 1357 out of 1564 (87.76%) RiPPs BGCs were also unknown. Overall, the distribution of known and unknown BGCs vary dramatically across the different kinds of metabolites in *Bacillus* species, in which the NRPs BGCs are the most abundant ones, comprising 2377 BGCs. Many of them are conserved and already characterized, but still a large number of unknown NRPs BGCs are identified for further study.

## Disscussion

*Bacillus* strains attract more and more attention due to their ability to produce hard, resistant endospores and antibiotics which have the potential to be used as biocontrol agents. In this study, We found that 34 *Bacillus* strains (out of 181 *Bacillus*-like strains) have antagonistic activity against six major tomato plant pathogens (*E. carotovora*, *P. syringae*, *R. solani*, *B. cinerea*, *V. dahliae,* and *P. infestans*). These results suggest that these strains have potentials for tomato diseases control. 16S rRNA gene sequences comparison of 34 *Bacillus* strains highlighted the diversity of *Bacillus* strains exhibiting antagonistic activity against plant pathogens, suggesting that strains from rhizosphere soil encounter different plant pathogens, resulting in the acquisition of antagonistic activity during evolution. The 34 *Bacillus* strains were clustered into three large clades according to their 16S rRNA sequences. By comparing the inhibition activity on the growth of each plant pathogen between these 34 strains, the most strongly inhibitory strains were closely related to *B. subtilis* and *B. velezensis,* suggesting that strains from *B. subtilis* and *B. velezensis* species possess strain-specific clusters of genes related to the biosynthesis of secondary metabolites, which play significant roles in pathogen suppression. In addition, among the 10 strains selected, two strains identified as *B. cabrialesii* BH5 and BH6 showed a growth reduction against all six major tomato plant pathogens (Fig. 1). This finding is the first record of this species that could exhibit antagonistic activity against tomato pathogens, which serves as a basis to further identify and characterize the interaction mechanisms between species *B. cabrialesii* and tomato pathogens.

Based on the genome mining, a total of 14 novel BGCs were revealed from the genomes of 10 sequenced strains. Five novel clusters were identified as NRPs and PKs-NRPs hybrid BGCs. These categories of BGCs encode non-ribosomally synthesized peptides synthetases (NRPSs) and hybrid polyketides synthases and non-ribosomally synthesized peptides synthetases (PKS-NRPSs), which are modular multienzymes. NRPSs construct peptides from amino acids and PKS-NRPSs construct hybrid molecules from acyl-CoA moieties together with amino acids [49]. So far, some known bioactive compounds identified from *Bacillus* strains belong to these modular biosynthetic compounds, such as surfactin [24], iturin [50], bacillomycin [24], fengcin [24] and difficidin [29]. All of them are antimicrobials which can be used for biocontrol in agriculture. As the economic loss caused by plant diseases is increasing in recent years, it is worthwhile to investigate the compounds produced by the five novel modular BGCs identified. Due to the antagonistic assays in vitro, we speculate some of them have antibacterial and (or) antifungal activities. This needs to be characterized by experiments in the future. Especially, the novel NRPs BGC identified from *B. velezensis* FH17 and TH16 possess a single heterocyclization (Cy) domain in the first module, which could modify cystine (Cys) to form a thiazoline ring. This domain first catalyzes amide bond formation, and then the intramolecular cyclodehydration between the side chain of the first amino acid (Cys) and the backbone carbonyl carbon takes place to form a thiazoline ring [51]. This ring is important for the structure and function of this lipopeptide product. So far, many well-known drugs for anti-microbial and anti-cancer activity exhibit thiazoline rings [52], such as Sulfathiazole (anti-microbial drug), Ritonavir (anti-retroviral drug), Tiazofurin (anti-neoplastic drug) and Abafungin (anti-fungal drug) [53]. These findings point to the potential anti-microbial activity of the compounds produced by this novel BGC in *B. velezensis* FH17 and TH16. Moreover, nine novel RiPPs BGCs were discovered. They are categorized into lanthipeptide I/II/III and lasso peptide BGCs. Lanthipeptides (also called lantibiotics for those with antibacterial activities) are ribosomally synthesized post-translationally modified peptides having thioether cross-linked amino acids, lanthionines, as a structural element [54]. They have potentials to be used as therapeutics. Subtilin, a lanthipeptide I, is one of the most studied bacteriocins from the *Bacillus* strains [15]. It is synthesized by *spa* BGC which is possessing strong antibiotic activities [55]. Mersacidin, is produced by *mrs* BGC in *Bacillus* sp. HIL Y-85/54728 which is belonging to lanthipeptide II. It has activity against Gram-positive bacteria including *Staphylococcus aureus*, *Streptococcus pneumoniae* and *Enterococcus faecium* [55]. However, to date, only Lanthipeptide I and II have been reported from *Bacillus* strains. Here, we report two class III lanthipeptide BGCs from *Bacillus* strains (Fig. 5f and g). Unlike other class III lathipeptides, the one from *B. velezensis* TH16 only consists of one lanthionine/labionin moiety instead of two. Both in vivo and in vitro maturation of this peptides have value to be investigated in the future. With an increasing number of genomes, more and more lanthipeptides BGCs are certainly discovered, but only a few of them have been characterized by experimental researches. Lasso peptides contain a macrocyclic linkage between an Asp or Glu side chain to the N- terminus of the core peptide. The C-terminal tail is threaded through the macrocycle, giving a lariat topology for which the lasso peptides are named [56]. Until now, some lasso peptides are reported with antimicrobial activity. i.e. lariatin [57], lassomycin [58] and microcin J25 [59]. However, we can not predict the compounds produced by these nine novel lanthipeptides and lasso peptides BGCs are antimicrobials or not, which also need to be investigated by further experiments. These 14 novel BGCs identified from *Bacilllus* stains appear to have different NRPs modules, precursor peptides or extra genes that might lead to modification on the biosynthetic pathway, changing the compound’s structure and their activity. Therefore, these novel BGCs are of great interest for antimicrobial activity and novel drug discovery. The expression of BGCs into biological active compounds, providing the real biocontrol effect of *Bacillus* sp., is still the main challenge. So far, it remains difficult to predict the exact compound products from genome sequence data only. Exploring the biosynthetic capacity of *Bacillus* sp. by genome mining in combination with advanced mass spectrometry techiniques provide feasible solution for future compounds identification and characterization.

The result of large-scale genome mining of bioactive potential of *Bacillus* shows the distribution of known and unknown BGCs varying dramatically across the different kinds of metabolites in *Bacillus* species. NRPs and PKs-NRPs BGCs in *Bacillus* species are encoding highly conserved known compounds as well as various novel variants. These findings are consistent with KirK J. Grubbs [60] reported. They found that majority of *Bacillus* natural products are comprised of a small set of highly conserved, well-distributed, known natural product compounds. The genus *Bacillus* is well known for the natural products with antibacterial and antifungal activities, which has a strong potential to be applied to agriculture for plant diseases control [61]. Therefore, novel antimicrobials discovery is in need of identification and characterization of novel BGCs in *Bacillus* strains.

## Conclusions

This work showed that 10 *Bacillus* and *Paenibacillus* strains, selected from 181 isolated *Bacillus*-like strains from the rhizosphere soil of healthy tomato plants and their tissues, have strong in vitro antagonistic activity against tomato bacterial, fungal and oomycetal pathogens. Based on genome mining, we identified a large number of BGCs from their genome sequences encoding known and unknown compounds, which form a great source for pharmaceutical compound discovery. Furthermore, a total of 14 novel BGCs were characterized in detail, including 2 NRPs, 3 PKs-NRPs hybrid and 9 RiPPs BGCs. In addition, from the large-scale bioinformatics analysis of the genomes from *Bacillus* genus, we found that NRPS and PKS-NRPS BGCs resources hidden in *Bacillus* species are frequently encoding highly conserved known compounds including surfactin, fengycin, bacillibactin, petrobactin, lichenysin and bacillaene.

## Methods

### Sample collection, Bacteria isolation, and culture conditions

Healthy tomato plants (cultivar: Boludo) and their rhizosphere soil were carefully collected during spring (February 2017) from tomatoes grown in a garden in the village of Roden in the Netherlands and Almería in Spain, which were given to us by company Koppert and with their consent. The bacterial isolation was performed as described previously [22]. Briefly, 1 g rhizosphere soil was suspended in 9 ml of 10 mM sterilized MgSO_4_ buffer. Then the suspension was diluted 10^3^–10^6^ times with 10 mM sterilized MgSO_4_ buffer. After dilution, all the samples were heat-treated at 80 °C for 15 min. and subsequently spread on Luria-Bertani (LB) agar plates. The plates were incubated at 28 °C for 24–48 h to obtain single colonies. For the isolation of endophytes, 1 g tomato leaves were surface-sterilized for 1 min. in 70% ethanol and 3 min. in 0.5% NaClO solution supplemented with one droplet Tween 80 per 100 ml solution and then rinsed 5 times with sterilized deionized water. After surface sterilization, the leaves were macerated in 9 ml of 10 mM sterilized MgSO_4_ buffer with a sterilized mortar to obtain the suspension. The following steps were the same as isolation from rhizosphere soil. The surface-sterilization process was checked by spreading aliquots of the last rinsing deionized water on LB agar plates (if no organism growth was observed after 7 days, surface sterilization was considered to be successful). All the isolated strains were stored in 25% glycerol solution at − 80 °C untill further investigation.

### Screening of antimicrobial activity

In vitro antagonistic activity assays were performed on dual culture plates as described before with slight modification [62]. All the isolated strains were screened against different bacterial, fungal and oomycetal plant pathogens, such as *E. carotovora*, *P. syringae*, *R. solani*, *B. cinerea*, *V. dahliae,* and *P. infestans*. All the isolated strains were tested with different pathogens in triplicates.

To test antibacterial activity, bacterial pathogens were mixed with pre-cooled LB agar media (around 55 °C) at a final concentration of 1X10^6^ cells/ml. Then the mixed media was poured into Petri dishes to obtain pathogen-fusion agar plates. 5 ul of 1X10^8^ cells/ml overnight culture of each isolated strain was inoculated at the center of plates. All the plates were incubated at 28 °C for 2 days before the clear halo surrounding the strain isolated was measured.

Antagonistic activity of isolated strains against *R. solani* was tested as follows. A 0.5-cm mycelium plug of 3-day-old *R. solani* was placed at the center of the 1/5th PDA plate, and 5 ul of 1X10^8^ cells/ml overnight culture of each isolated strain was inoculated at a distance of 2 cm from the fungus. Plates were incubated at 28 °C for 3 days and inhibition of fungal growth was recorded as the diameter of the inhibition zone (mm). As a control, LB media was used in place of the bacterial suspension.

Antagonistic activity determination of isolated strains against *B. cinerea*, *V. dahliae,* and *P. infestans* was performed similarly as the antibacterial activity assay. Spores of *B. cinerea* and *V. dahliae* were collected respectively from 7-day-old and 20-day-old PDA plates with sterilized Mili-Q water by washing the mycelium. All the spores were counted using a Thoma chamber and were then mixed into 1/5th PDA media and adjusted to 1X10^7^ spores/ml. In addition, sporangia of *P. infestans* were harvested by washing the 30-day-old RSA plates with sterilized mili-Q water and then counted and mixed into 1/5th PDA media at the final concentration of 4000 sporangia/ml. Before mixing, the sporangia suspension was stimulated to release zoospores by chilling for 1–3 h at 4 °C. Subsequently, the mixed 1/5th PDA media was poured into Petri dishes. 5 ul of 1X10^8^ cells /ml overnight culture of each isolated strain was inoculated at the center of plates. All the plates were incubated at 28 °C. The clear halo surrounding each isolated strain was monitored and measured depending on the pathogens’ growth rate.

### Identification of bacterial strains

The isolated strains showing activity against pathogens were identified through partial 16S rRNA sequence homology analysis. The polymerase chain reaction (PCR) was carried out with bacterial-specific 16S rRNA primers 27F (5′-AGAGTTTGATCMTGGCTCAG-3′) and 1492R (5′-CGGTTACCTTGTTACGACTT-3′) using genomic DNA as templates. The PCR (50 μl) contained following final concentrations: 10 μl of five-fold high fidelity Phusion buffer (Thermo Scientific), 0.4 mM of each of the four deoxynucleoside triphosphates (Thermo Scientific), 0.4 μM of each primer, 0.5 U of Phusion high fidelity DNA polymerase (Thermo Scientific), and ∼50 ng of the isolated genomic DNA. The following thermal cycling scheme was used: initial denaturation at 98 °C for 3 min, 30 cycles of denaturation at 98 °C for 15 s, annealing at 56 °C for 15 s, extension at 72 °C for 50 s, followed by an additional extension step at 72 °C for 7 min. The PCR products were then purified with a NucleoSpin Gel and PCR Clean-up kit (Macherey-Nagel) and sequenced at Macrogen Inc. The resulting 16S rRNA gene sequences were compared in a BLAST search to the NCBI database. Phylogenetic analysis was performed using the MEGA software package (Version X) [63]. The relationships between sequences were analyzed using the neighbor-joining method. Bootstrap analysis was used to evaluate the tree topology of the neighbor-joining data by analyzing 1000 randomized data sets. The phylogenetic tree was visualized in iTOL version 4.4.2 [64].

### Genome sequencing and phylogenetic analysis

Genomic DNA of isolates was extracted with a GenElute Bacterial Genomic DNA kit (Sigma) according to the manufacturer’s protocol and sequenced at GATC Biotech (Germany) with an Illumina HiSeq sequencing system. The draft genomes were assembled and deposited in GeneBank. For the classification of species affiliations, dDDH and ANI values were calculated using the web tools TYGS [65] and JSpeciesWS [66] respectively. Two genomes belonging to the same species would have a dDDH of at least 70%, which corresponds to at least 95% ANI [23]. The whole genomes were then compared with other reference genomes using the GEGENEES tool [67] based on a fragmented nucleotides alignment with a setting of 200/100. A phylogenetic tree was generated with iTOL version 4.4.2 [64] and Splitstree [68].

### Antimicrobial compounds mining among 10 isolated strains

For identification of biosynthesis gene clusters (BGCs) in isolated strains, each draft genome was assembled into a pseudomolecule using Medusa web server (http://combo.dbe.unifi.it/medusa) [69] based on multiple closely related strains as references. And then all the pseudomolecules were send to antiSMASH [70] and BAGEL4 [71] for BGCs mining.

### Metabolite BGC network analysis among *Bacillus* strains

The complete genomes of 555 *Bacillus* strains from 60 species of *Bacillales* were downloaded from Genebank and analyzed by antiSMASH 5.0 [70]. The similarity network between BGCs was calculated with BiG-SCAPE (https://git.wageningenur.nl/medema-group/BiG-SCAPE) [72]. A program that constructs sequence similarity networks of BGCs and groups them into Gene Cluster Families (GCFs) [72]. To visualize, the distance matrix between BGCs generated by BiG-SCAPE was exported and annotated in Cytoscape v3.7.0 (http://www.cytoscape.org/) [73]. Default parameters were used for all software unless noted.

## Supplementary Information

**Additional file 1: Table S1.** Digital DNA-DNA Hybridization (dDDH) values (in upper triangle) and Average Nucleotide Identity (ANI) values (in lower triangle) amongst different strains. **Table S2.** All BGCs (including known and unknown) found in each genome of selected strains.

## Data Availability

The whole genome data are available at DDBJ/EMBL/GenBank under the bioproject accession PRJNA503984 (https://www.ncbi.nlm.nih.gov/bioproject/PRJNA503984/).

## Abbreviations

PGPR: Plant growth promoting rhizobacteria
BGCs: Biosynthetic gene clusters
ISR: Inducing systemic resistance
NRPSs: Non-ribosomally synthesized peptides synthetases
PKSs: Polyketides synthases
NRPs: Non-ribosomally synthesized peptides
PKs: Polyketides
PKS-NRPSs: Hybrid polyetides synthases and non-ribosomally synthesized peptides synthetases
RiPPs: Ribosomally synthesized and post-translationally modified peptides
PTMs: Posttranslational modifications
C: Condensation
A: Adenylation
T: Thiolation
E: Epimerization
TE: Thioesterase
KS: Ketosynthase
AT: Acyltransferase
ACP: Acyl carrier protein
TD: Reductase
DHt: Dehydratase domain variant
Cy: Heterocyclization
