# Supplementary material for: Antimicrobial activity screening of rhizosphere soil bacteria from tomato and genome-based analysis of their antimicrobial biosynthetic potential
Source: BMC Genomics. 2021 Jan 7;22:29. doi: 10.1186/s12864-020-07346-8 (PMC7789753; doi:10.1186/s12864-020-07346-8)
Supplement: Supplementary file 1 — Additional file 1: Table S1. Digital DNA-DNA Hybridization (dDDH) values (in upper triangle) and Average Nucleotide Identity (ANI) values (in lower triangle) amongst different strains. Table S2. All BGCs (including known and unknown) found in each genome of selected strains. [file 12864_2020_7346_MOESM1_ESM.docx]

**Supplementary data**

**Table S1**. Digital DNA-DNA Hybridization (dDDH) values (in upper triangle) and Average Nucleotide Identity (ANI) values (in lower triangle) amongst different strains

|  | *Bacillus cabrialessi* BH5 | *Bacillus cabrialessi* BH6 | *Bacillus subtilis* DH12 | *Bacillus subtilis* EH2 | *Bacillus subtilis* EH5 | *Bacillus subtilis* EH11 | *Bacillus subtilis* NCD-2 | *Bacillus cabrialessi* TE3 | *Bacillus subtilis* Bsn5 | *Bacillus velezensis* FH17 | *Bacillus velezensis* TH16 | *Bacillus velezensis* DSM7 | *Bacillus velezensis* FZB42 | *Bacillus velezensis* SQR9 | *Bacillus endophyticus* FH5 | *Bacillus endophyticus* KCTC 13922 | *Paenibacillus* sp. EDO6 | *Paenibacillus polymyxa* E681 | *Paenibacillus polymyxa* SC2 | *Paenibacillus xylanexedens* PAMC 22703 |
| --- | --- | --- | --- | --- | --- | --- | --- | --- | --- | --- | --- | --- | --- | --- | --- | --- | --- | --- | --- | --- |
| *Bacillus cabrialessi* BH5 |  | **100.00** | **47.20** | **47.40** | **47.30** | **47.20** | **47.40** | **73.40** | **47.40** | **20.60** | **20.70** | **20.70** | **20.80** | **20.70** | **20.60** | **20.60** | **21.20** | **26.60** | **26.10** | **26.50** |
| *Bacillus cabrialessi* BH6 | 100.00 |  | **47.10** | **47.40** | **47.30** | **47.10** | **47.40** | **73.40** | **47.40** | **20.60** | **20.70** | **20.70** | **20.80** | **20.70** | **20.50** | **20.60** | **21.10** | **26.50** | **26.10** | **26.50** |
| *Bacillus subtilis* DH12 | 91.94 | 91.94 |  | **85.30** | **91.40** | **99.80** | **85.00** | **47.60** | **87.70** | **20.40** | **20.40** | **20.50** | **20.50** | **20.50** | **20.80** | **20.50** | **21.40** | **26.10** | **26.10** | **27.30** |
| *Bacillus subtilis* EH2 | 92.08 | 92.09 | 98.17 |  | **85.80** | **85.30** | **84.70** | **47.80** | **86.90** | **20.40** | **20.40** | **20.50** | **20.60** | **20.50** | **21.90** | **20.80** | **21.40** | **25.90** | **26.00** | **27.30** |
| *Bacillus subtilis* EH5 | 91.88 | 91.88 | 98.81 | 98.04 |  | **91.40** | **84.80** | **47.60** | **86.70** | **20.30** | **20.40** | **20.50** | **20.60** | **20.50** | **20.40** | **20.80** | **21.40** | **26.00** | **26.00** | **27.30** |
| *Bacillus subtilis* EH11 | 91.93 | 91.93 | 99.96 | 98.03 | 98.86 |  | **84.90** | **47.60** | **87.70** | **20.40** | **20.40** | **20.50** | **20.60** | **20.50** | **20.80** | **20.50** | **21.50** | **26.30** | **26.30** | **27.50** |
| *Bacillus subtilis* NCD-2 | 92.07 | 92.07 | 98.14 | 98.01 | 98.07 | 98.14 |  | **47.80** | **84.70** | **20.70** | **20.70** | **20.90** | **20.80** | **20.90** | **24.70** | **24.10** | **26.20** | **27.20** | **27.50** | **28.50** |
| *Bacillus cabrialessi* TE3 | 96.60 | 96.60 | 92.20 | 92.12 | 92.22 | 92.20 | 92.17 |  | **47.80** | **20.40** | **20.60** | **27.40** | **28.70** | **28.10** | **20.90** | **21.40** | **21.20** | **26.70** | **26.10** | **26.60** |
| *Bacillus subtilis* Bsn5 | 91.98 | 91.99 | 98.46 | 98.21 | 98.37 | 98.46 | 97.96 | 92.20 |  | **20.40** | **20.50** | **20.80** | **20.70** | **20.70** | **25.10** | **23.90** | **26.20** | **27.70** | **27.90** | **28.90** |
| *Bacillus velezensis* FH17 | 76.42 | 76.42 | 76.12 | 76.06 | 76.08 | 76.11 | 76.17 | 76.22 | 76.06 |  | **84.70** | **55.50** | **85.30** | **84.20** | **21.60** | **21.60** | **26.00** | **28.40** | **28.70** | **32.00** |
| *Bacillus velezensis* TH16 | 76.47 | 76.45 | 76.05 | 76.14 | 76.07 | 76.05 | 76.21 | 76.27 | 76.07 | 98.01 |  | **55.40** | **90.10** | **85.90** | **22.40** | **21.70** | **26.80** | **28.50** | **28.70** | **32.60** |
| *Bacillus velezensis* DSM7 | 76.74 | 76.74 | 76.37 | 76.40 | 76.35 | 76.37 | 76.39 | 76.50 | 76.39 | 93.85 | 93.82 |  | **56.20** | **55.60** | **26.20** | **24.80** | **31.80** | **29.20** | **29.90** | **31.20** |
| *Bacillus velezensis* FZB42 | 76.72 | 76.72 | 76.31 | 76.34 | 76.31 | 76.31 | 76.40 | 76.53 | 76.25 | 98.14 | 98.17 | 93.36 |  | **86.80** | **26.70** | **25.80** | **30.00** | **29.50** | **30.00** | **31.70** |
| *Bacillus velezensis* SQR9 | 76.39 | 76.39 | 76.12 | 76.16 | 76.08 | 76.10 | 76.17 | 76.16 | 76.04 | 97.72 | 98.00 | 93.08 | 98.06 |  | **23.10** | **23.90** | **29.20** | **29.00** | **29.40** | **31.30** |
| *Bacillus endophyticus* FH5 | 67.01 | 67.00 | 67.11 | 67.14 | 67.13 | 67.11 | 67.15 | 67.06 | 67.08 | 66.64 | 66.48 | 66.61 | 66.66 | 66.61 |  | **73.40** | **20.00** | **25.50** | **26.10** | **26.10** |
| *Bacillus endophyticus* KCTC 13922 | 67.24 | 67.24 | 67.27 | 67.23 | 67.36 | 67.27 | 67.29 | 67.27 | 67.28 | 66.59 | 66.56 | 66.60 | 66.64 | 66.53 | 96.35 |  | **20.50** | **25.70** | **25.90** | **26.00** |
| *Paenibacillus* sp. EDO6 | 64.51 | 64.51 | 64.34 | 64.35 | 64.34 | 64.33 | 64.38 | 64.51 | 64.31 | 64.32 | 64.37 | 64.39 | 64.36 | 64.44 | 64.74 | 64.68 |  | **25.20** | **25.30** | **57.60** |
| *Paenibacillus polymyxa* E681 | 65.08 | 65.08 | 65.27 | 65.25 | 65.21 | 65.27 | 65.29 | 65.29 | 65.26 | 65.05 | 64.99 | 64.99 | 65.07 | 65.06 | 65.62 | 65.61 | 70.20 |  | **39.80** | **70.06** |
| *Paenibacillus polymyxa* SC2 | 65.37 | 65.37 | 65.47 | 65.51 | 65.54 | 65.47 | 65.53 | 65.58 | 65.55 | 65.40 | 65.46 | 65.34 | 65.38 | 65.38 | 65.88 | 65.94 | 70.16 | 89.49 |  | **69.94** |
| *Paenibacillus xylanexedens* PAMC 22703 | 65.16 | 65.16 | 65.20 | 65.19 | 65.17 | 65.20 | 65.13 | 65.18 | 65.14 | 65.21 | 65.19 | 65.31 | 65.24 | 65.27 | 65.50 | 65.57 | 93.88 | 70.06 | 69.94 |  |

**Table S2**. All BGCs (including known and unknown) found in each genome of selected strains

| Strains | cluster | Size(bp) | Type | Compounds |
| --- | --- | --- | --- | --- |
| *B. cabrialesii* BH5 | 1 | 20264 | Bacteriocin | Rhizoctin A ( partial) |
|  | 2 | 63861 | NRPS | Surfactin |
|  | 3 | 19891 | Terpene | unknown |
|  | 4 | 66797 | NRPS | unknown |
|  | 5 | 114089 | Hybrid NRPS/PKS | Bacillaene |
|  | 6 | 111369 | NRPS | Fengycin |
|  | 7 | 21590 | Terpene | unknown |
|  | 8 | 27899 | Bacterocin | Staphylococcin C55 |
|  | 9 | 49743 | NRPS | Bacillibactin |
|  | 10 | 21612 | Bacteriocin | Subtilosin A |
|  | 11 | 41419 | Other | bacilysin |
|  | 12 | 41098 | Type III PKS | unknown |
| *B. cabrialesii* BH6 | 1 | 20264 | Bacteriocin | Rhizoctin A ( partial) |
|  | 2 | 63861 | NRPS | Surfactin |
|  | 3 | 19891 | Terpene | unknown |
|  | 4 | 66797 | NRPS | unknown |
|  | 5 | 114089 | Hybrid NRPS/PKS | Bacillaene |
|  | 6 | 111269 | NRPS | Fengycin |
|  | 7 | 21590 | Terpene | unknown |
|  | 8 | 27889 | Bacterocin | Staphylococcin C55 |
|  | 9 | 49743 | NRPS | Bacillibactin |
|  | 10 | 21612 | Bacteriocin | Subtilosin A |
|  | 11 | 41419 | Other | bacilysin |
|  | 12 | 41098 | Type III PKS | unknown |
| *B. subtilis* DH12 | 1 | 62295 | NRPS | Surfactin |
|  | 2 | 20525 | Terpene | unknown |
|  | 3 | 49742 | NRPS | Bacillibactin |
|  | 4 | 114764 | Hybrid NRPS/PKS | Bacillaene |
|  | 5 | 77493 | NRPS | Fengycin |
|  | 6 | 41764 | NRPS | Fengycin (partial) |
|  | 7 | 21899 | Terpene | unknown |
|  | 8 | 41098 | Type III PKS | unknown |
|  | 9 | 26097 | Bacteriocin | unknown |
|  | 10 | 26226 | Bacteriocin | Subtilin |
|  | 11 | 21612 | Bacteriocin | Subtilosin A |
|  | 12 | 41419 | Other | Bacilysin |
| *B. subtilis* EH2 | 1 | 41419 | Other | Bacilysin |
|  | 2 | 21612 | Bacterocin | SubtilosinA |
|  | 3 | 49267 | NRPS | Bacillibactin |
|  | 4 | 40889 | Type III PKS | unknown |
|  | 5 | 20855 | Terpene | unknown |
|  | 6 | 81195 | NRPS | Fengycin |
|  | 7 | 114800 | Hybrid NRPS/PKS | Bacillaene |
|  | 8 | 20807 | Terpene | unknown |
|  | 9 | 26330 | Bacteriocin | unkown |
|  | 10 | 65099 | NRPS | Surfactin |
| *B. subtilis* EH5 | 1 | 26107 | Bacteriocin | unknown |
|  | 2 | 41419 | Other | Bacilysin |
|  | 3 | 21612 | Bacteriocin | Subtilosin A |
|  | 4 | 49057 | NRPS | Bacillibactin |
|  | 5 | 23117 | Bacteriocin | unknown |
|  | 6 | 40666 | Type III PKS | unknown |
|  | 7 | 21548 | Terpene | unknown |
|  | 8 | 80494 | NRPS | Fengycin |
|  | 9 | 114763 | Hybrid NRPS/PKS | Bacillaene |
|  | 10 | 20804 | Terpene | unknown |
|  | 11 | 64847 | NRPS | Surfactin |
| *B. subtilis* EH11 | 1 | 62285 | NRPS | Surfactin |
|  | 2 | 20525 | Terpene | unknown |
|  | 3 | 26097 | Bacteriocin | unknown |
|  | 4 | 114764 | Hybrid NRPS/PKS | Bacillaene |
|  | 5 | 91208 | NRPS | Fengycin |
|  | 6 | 42435 | NRPS | Fengycin (partial) |
|  | 7 | 21899 | Terpene | unknown |
|  | 8 | 41098 | Type III PKS | unknown |
|  | 9 | 49742 | NRPS | Bacillibactin |
|  | 10 | 26226 | Bacteriocin | Subtilin |
|  | 11 | 21612 | Bacteriocin | Subtilosin A |
|  | 12 | 41419 | Other | Bacilysin |
| *B. endophyticus* FH5 | 1 | 23854 | Bacteriocin (lsso peptide) | unknown |
|  | 2 | 58658 | NRPS | Bacillibactin |
|  | 3 | 65159 | NRPS | unknown |
|  | 4 | 30327 | Terpene | unknown |
|  | 5 | 82686 | Hybrid NRPS/PKS | unknown |
|  | 6 | 18284 | Terpene | unknown |
|  | 7 | 41089 | Type III PKS | unknown |
|  | 8 | 20819 | Terpene | unknown |
|  | 9 | 29091 | Bacteriocin | unknown |
|  | 10 | 23546 | Bacteriocin (LAP) | unknown |
| *B. velezensis* FH17 | 1 | 65263 | NRPS | Surfactin |
|  | 2 | 40885 | Other | unknown |
|  | 3 | 22183 | Bacteriocin (LAP) | Plantazolicin |
|  | 4 | 41245 | PKS-like | unknown |
|  | 5 | 17275 | Terpene | unknown |
|  | 6 | 87810 | trans AT-PKS | Macrolactin H |
|  | 7 | 64823 | NRPS | unknown |
|  | 8 | 21884 | Terpene | unknown |
|  | 9 | 136324 | NRPS | Fengycin |
|  | 10 | 41101 | Type III PKS | unknown |
|  | 11 | 106174 | Hybrid NRPS/PKS | Difficidin |
|  | 12 | 50503 | NRPS | Bacillibactin |
|  | 13 | 108797 | Hybrid NRPS/PKS | Bacillaene |
|  | 14 | 41419 | Other | Bacilysin |
|  | 15 | 2458 | NRPS | unknown |
| *B. velezensis* TH16 | 1 | 106180 | Hybrid NRPS/PKS | Difficidin |
|  | 2 | 64842 | NRPS | Surfactin |
|  | 3 | 17419 | Terpene | unknown |
|  | 4 | 86391 | trans AT-PKS | Macrolactin H |
|  | 5 | 109604 | Hybrid NRPS/PKS | Bacillaene |
|  | 6 | 71730 | NRPS | Fengycin |
|  | 7 | 41245 | PKS-like | unknown |
|  | 8 | 41101 | Type III PKS | unknown |
|  | 9 | 22616 | Bacteriocin | unknown |
|  | 10 | 50949 | NRPS | Bacillibactin |
|  | 11 | 41419 | Other | Bacilysin |
|  | 12 | 68418 | NRPS | unknown |
| *Paenibacillus* sp. EDO6 | 1 | 20873 | Terpene | unknown |
|  | 2 | 34652 | Type III PKS | unknown |
|  | 3 | 49099 | NRPS | Bacillibactin |
|  | 4 | 22745 | Bacteriocin | unknown |
|  | 5 | 17722 | Other | unknown |
|  | 6 | 10243 | Bacteriocin | unknown |
|  | 7 | 23968 | Bacteriocin (lasso peptide) | unknown |
|  | 8 | 41210 | NRPS-like | unknown |
|  | 9 | 26366 | Bacteriocin | unknown |
|  | 10 | 41158 | Type III PKS | unknown |
|  | 11 | 22961 | Bacteriocin (LAP) |  |
|  | 12 | 60514 | trans-AT PKs-NRPs hybrid | unknown |
|  | 13 | 83731 | trans-AT PKs-NRPs hybrid | unknown |
|  | 14 | 81118 | NRPS | Polymyxin |
